# A morphological study on the sphenoid bone ligaments’ ossification pattern

**DOI:** 10.1007/s00276-023-03226-4

**Published:** 2023-08-07

**Authors:** Maria Piagkou, Aliki Fiska, George Tsakotos, George Triantafyllou, Constantinus Politis, Christos Koutserimpas, Janusz Skrzat, Lukasz Olewnik, Nicole Zielinska, Athina Tousia, Michael Kostares, Trifon Totlis, Anastasia Triantafyllou, Katerina Al Nasraoui, Vasilios Karampelias, Christos Tsiouris, Konstantinos Natsis

**Affiliations:** 1 Department of Anatomy, School of Medicine, Faculty of Health Sciences, National and Kapodistrian University of Athens, 75 Mikras Asias Str., Goudi, 11527 Athens, Greece; 2 Laboratory of Anatomy, School of Medicine, Democritus University of Thrace, Alexandroupolis, Greece; 3 Department of Imaging and Pathology, Faculty of Medicine, KU Leuven, University Hospitals Leuven, Leuven, Belgium; 4 Department of Orthopaedics and Traumatology, “251” Hellenic Air Force General Hospital of Athens, Athens, Greece; 5 Department of Anatomy, Jagiellonian University Medical College, Krakow, Poland; 6 Department of Anatomical Dissection and Donation, Medical University of Lodz, Lodz, Poland; 7 Department of Anatomy and Surgical Anatomy, School of Medicine, Faculty of Health Sciences, Aristotle University of Thessaloniki, Thessaloniki, Greece

**Keywords:** Ligament, Ossification, Skull, Sphenoid bone, Pterygospinous, Pterygoalar, Caroticoclinoid, Interclinoid, Sella

## Abstract

**Purpose:**

The sphenoid bone (SB) extracranial ligaments (ECRLs) are the pterygoalar and pterygospinous ligaments (PTAL and PTSL) that are located at the SB lateral pterygoid plate, and inferior to the foramen ovale (FO). Their ossification may affect the mandibular nerve’s distribution. The intracranial ligaments’ (ICRLs) ossification (the caroticoclinoid ligament—CCLL, the anterior and posterior interclinoid ligaments—AICLL and PICLL) may impede the approaches to the sella.

This study highlights the incidence of the ossified ECRLs and ICRLs location, their type (partial, or complete), considering laterality, gender, age, and ligaments’ simultaneous presence.

**Methods:**

The sample consisted of 156 Greek adult dried skulls of both genders and variable age.

**Results:**

Ossified ligaments were identified in 57.05%, predominantly extracranially (42.31%, *P* = 0.003). ECRLs were predominantly identified unilaterally (30.13%, *P* < 0.001). The majority of the ossified ICRLs were predominantly identified in male skulls (31.1%, *P* = 0.048) and the majority of the ECRLs (52.8%, *P* = 0.028) were predominantly identified at the age of 60 years and above. The PTAL was the most ossified (32.69%), followed by the CCLL (24.36%), the PTSL (16.03%), the PICLL (6.41%), and the AICLL (4.49%).

**Conclusions:**

Detailed knowledge of the SB morphology and ligaments’ ossification extent is essential to improve the technique of the FO percutaneous approach, and sellar approaches, to minimize complications.

## Introduction

**Fig. 1:**
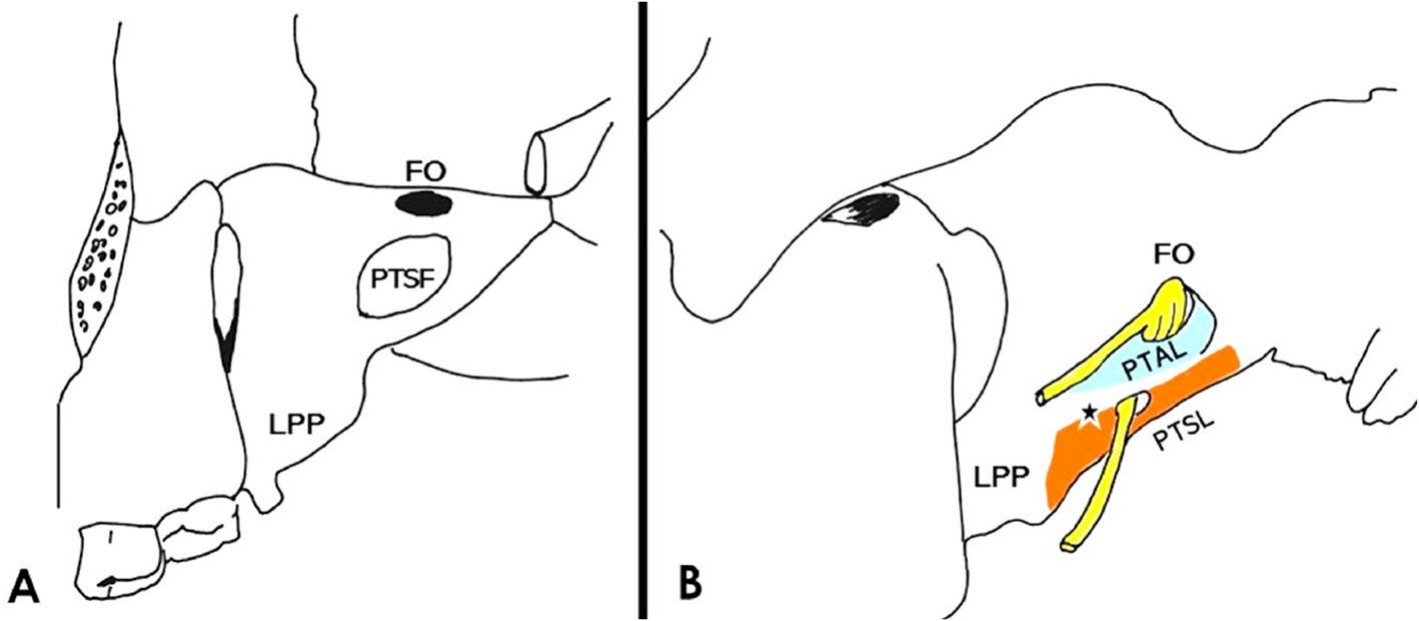
**Α** Extracranially, in the infratemporal fossa, the ossification of the pterygospinous ligament (PTSL) attached to the lateral pterygoid plate (LPP) inferior border and the spine of the sphenoid bone. The pterygospinous bar (PTSB) and the resulting homonymous foramen (PTSF, black asterisk) below the foramen ovale (FO). **Β** The ossified PTSL (orange part) and the pterygoalar ligament (PTAL, light blue part) into the PTSB and pterygoalar bar (PTAB) and their proximity to the FO and the mandibular nerve distribution (color figure online)

**Fig. 2:**
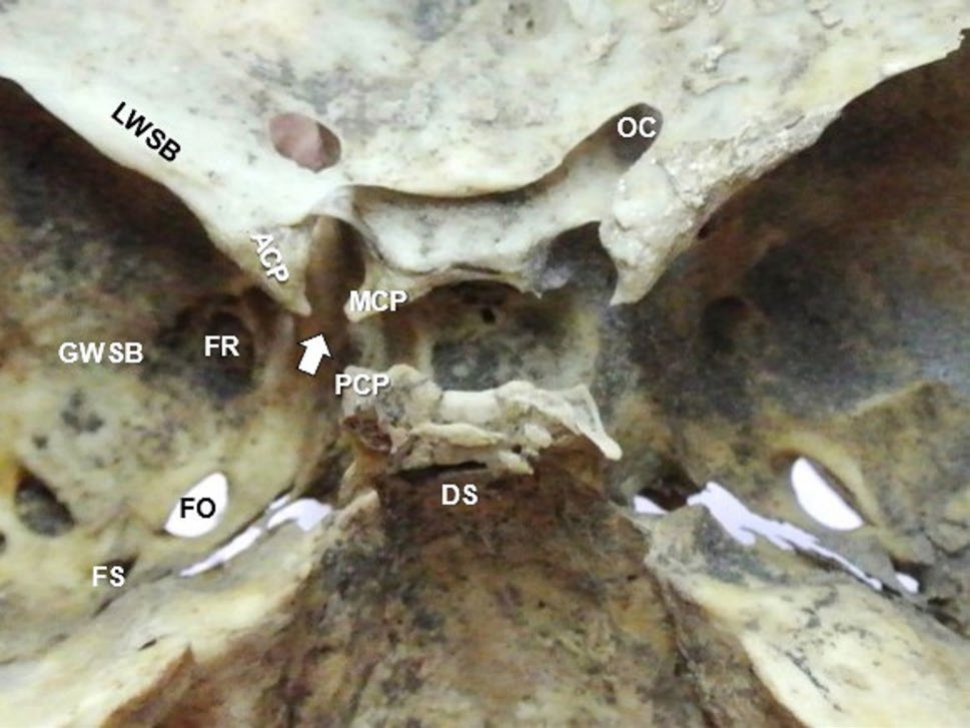
Skull intracranial view, depicting the sella with its ligaments between the anterior clinoid process (ACP), the middle clinoid process (MCP), and the posterior clinoid process (PCP). The ligament connecting ACP–MCP depicts the caroticoclinoid ligament (CCLL, white arrow), the ligament between MCP and PCP depicts the posterior interclinoid ligament (PICLL) and the ligament ACP–PCP depicts the anterior interclinoid ligament (AICLL), OC—optic canal, and DS—dorsum sella, FR—foramen rotundum, FO—foramen ovale, FS—foramen spinosum, LWSB—lesser wings of the sphenoid bone, GWSB—greater wings of the sphenoid bone

Ligaments are dense, fibrous connective structures, and their ossification is frequently identified in various parts of the human body, as an age-dependent process [35]. In the sphenoid bone (SB) of the skull, both extracranial and intracranial ligaments (ECRLs and ICRLs) may be partially or completely ossified, unilaterally or bilaterally resulting in extracranial and intracranial bars (ECRBs and ICRBs). In the infratemporal fossa, two ECRLs (the pterygoalar and pterygospinous ligament, PTAL and PTSL) are identified around the SB lateral pterygoid plate (LPP), and inferior to the foramen ovale (FO). The PTAL, a thin and dense-fibrous bundle, extends from the root of the LPP to the inferior surface of the greater sphenoidal wing [10]. It lies laterally or beneath the FO, dividing it into two parts, in a horizontal axis [8, 9]. The PTSL extends from the pterygospinous process of the LPP to the SB angular spine and lies either below or medial to the FO in a vertical axis [8, 9]. PTSL ossification in a pterygospinous bar (PTSB) affects the mandibular nerve’s distribution pattern, passing through the FO (Fig. 1). In the middle cranial fossa, three ICRLs are identified around the sella, area of the clinoid processes (CPs): 1) the caroticoclinoid ligament (CCLL) located between the anterior and middle clinoid process (ACP and MCP), 2) the anterior interclinoid ligament (AICLL) located between the ACP and posterior clinoid process (PCP), and 3) the posterior interclinoid ligament (PICLL) located between the MCP and PCP (Fig. 2). The CCLL complete ossification results in the caroticoclinoid bar (CCLB) and the homonymous foramen that is penetrated by the internal carotid artery (ICA). The PICLL complete ossification results in the posterior interclinoid bar (PICLB) and foramen that gives passage to the lateral part of the circular sinus [27]. Skull base imaging by three-dimensional computed tomography (3DCT) [30, 61] is essential to identify the SB extracranial and intracranial ossification pattern and their extent. Focusing on the SB clinical importance, and the lack of evidence concerning the simultaneous investigation of the ECRLs and ICRLs anatomy, this study highlights the incidence of the exact location (extracranial and intracranial) of the SB ligaments’ ossification (PTALB, PTSB, CCLB, AICLB, and PICLB), their type (partial, or complete) in dried skulls, taking into consideration laterality (unilateral or bilateral side of occurrence), the gender, and the age. The simultaneous presence of the ECRBs and ICRBs was also calculated.

## Materials and methods

One hundred and fifty-six (156) Greek adult dried skulls were obtained from the Anatomy and Surgical Anatomy Department of the Aristotle University of Thessaloniki and the Anatomy Department of the National and Kapodistrian University of Athens. All skulls were bilaterally observed (312 sides) for the presence of the two ECRBs (PTSB and PTAB) and three ICRBs (CCLB, AICLB, and PICLB). The ossification type (partial or complete) was recorded according to the side of appearance (unilateral—right or left side or bilateral side). Partial ossification was considered the ossification extended at least 25% of the ligament’s length. Concerning the ICRLs, complete ossification was considered the ossification of the entire ligament from the tip of the one CP to the other CP tip, i.e., from the ACP to the MCP (CCLB), from the ACP to the PCP (AICLB), and from the MCP to the PCP (PICLB). As demographics were not available for all samples, among the 156 skulls, data regarding gender were known for 129 (78 male and 51 female) skulls, and age was available for 110 skulls. The sample of 110 skulls was further subclassified into three age groups: 1st group—20–39 years (33 skulls), 2nd group—40–59 years (24 skulls), and 3rd group—60 years and above (53 skulls). No skulls showed evidence of obvious trauma or pathological condition. Skulls with broken parts in the investigated area were excluded.

*Statistical analysis* was performed using IBM SPSS Statistics version 28.0. Gender dimorphism and age distribution were investigated using the chi-square test, while side asymmetry and differences between ligaments were further examined with the McNemar test. A P-value of less than 0.05 was considered significant.

## Results

**Fig. 3:**
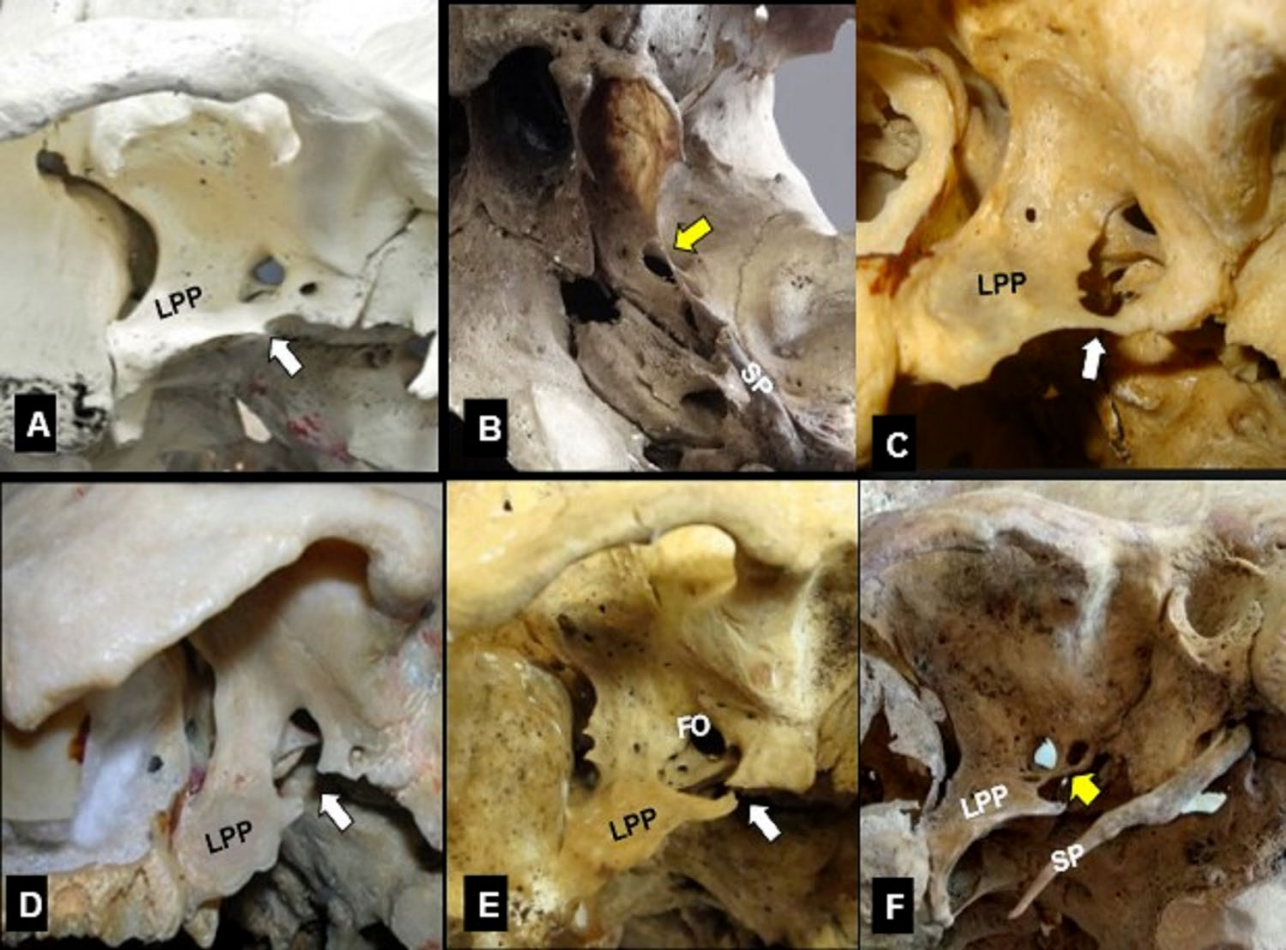
**Α–F** Extracranial skull base view, depicting the infratemporal fossa with the lateral pterygoid plate (LPP) and the ossified pterygospinous ligament (PTSL, white arrows) and pterygoalar ligament (PTAL, yellow arrows) below the foramen ovale (FO). **A**, **C** The complete pterygospinous bar (PTSB) and **B**, **F** The complete pterygoalar bar (PTAB). **D**, **E** The partial PTSB, SP-styloid process. **F** Two pterygoalar foramina (PTAF) anterolaterally and anteromedially to the yellow arrow (color figure online)

**Fig. 4:**
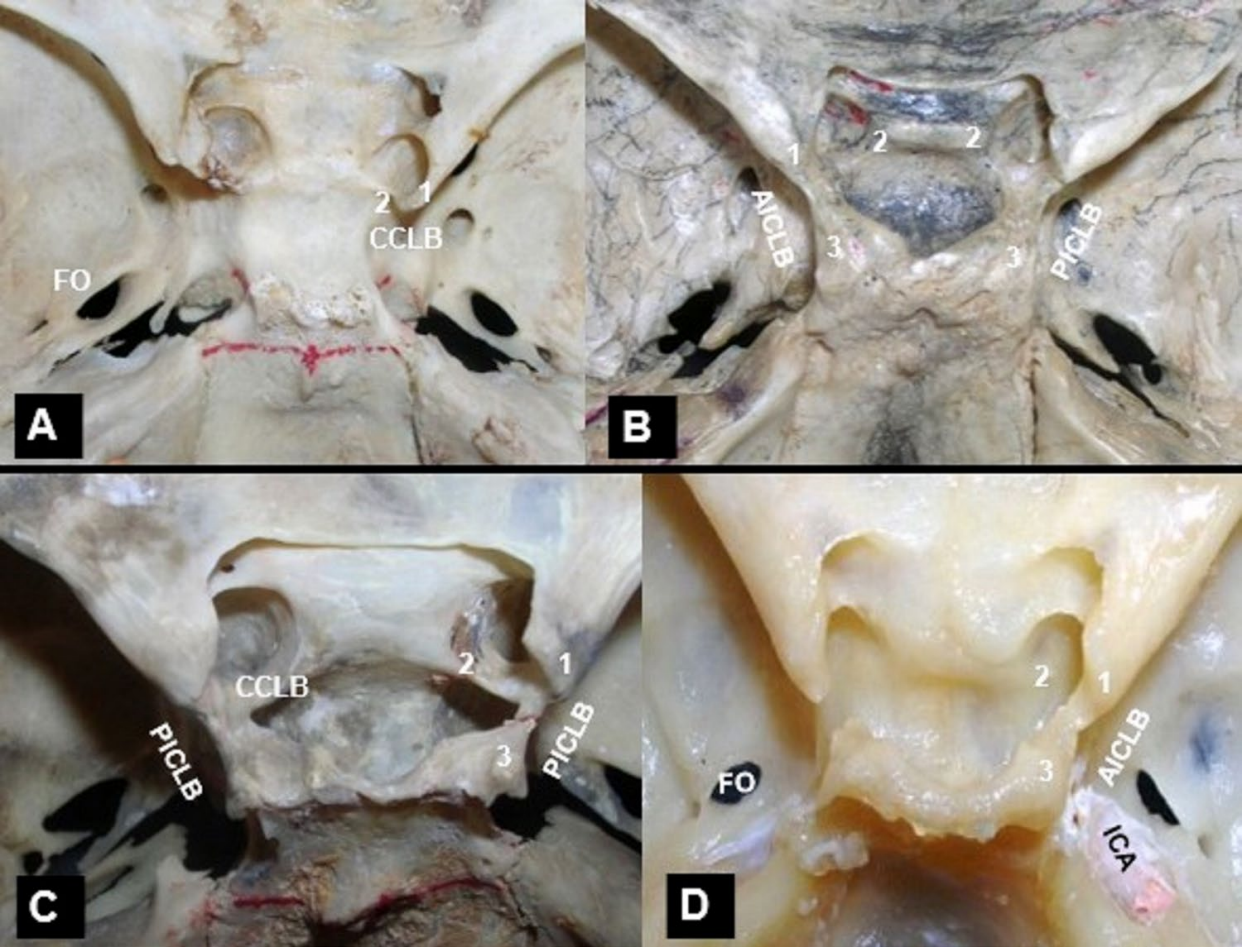
Intracranial view of the sella with the identified ossified intracranial ligaments (ICRLs) between the anterior (1), and middle (2) clinoid processes and the posterior clinoid process (3). **Α**, **C** The caroticoclinoid bar (CCLB, 1–2), **B**, **D** the anterior interclinoid bar (AICLB, 1–3) and **B**, **C** the posterior interclinoid bar (PICLB, 2–3). ICA—internal carotid artery, FO-foramen ovale. **C** A mixed ossification pattern bilaterally and **B** Unilaterally (right side)

Ossified ligaments were identified in 57.05% of the skulls (89/156), isolated ECRBs or ICRBs in 46.15% (72 skulls), and combined ECRBs and ICRBs in 10.89% (17 skulls) (Tables 1, 2). The ossified ligaments’ distribution by (extracranial or intracranial) location and ossification pattern (isolated or mixed) is summarized in Tables 1, 2. ECRBs were predominantly identified in 42.31% (66 skulls, *P* = 0.003, in 47 skulls unilaterally and in 19 skulls bilaterally) (Fig. 3) compared to the ICRBs in 25.64% (40 skulls, in 25 skulls bilaterally and in 15 skulls unilaterally) (Fig. 4). The ossified ligaments’ distribution by unilateral or bilateral occurrence and by side (right or left) is summarized in Tables 3, 4. Although side symmetry was identified separately in every bar’s presence, a right-side predominance was identified in interclinoid bars’ (ICLBs) existence (11.85%, 37 sides, *P* = 0.035), compared to the left-side (8.97%, 28 sides) (Tables 3, 4). The most ossified ligament was the extracranial PTAL (in 32.69%, 51 skulls), followed by the intracranial CCLL (in 24.36%, 38 skulls), the extracranial PTSL (in 16.03%, 25 skulls), and the intracranial PICLL (in 6.41%, 10 skulls) and AICLL (in 4.49%, 7 skulls). The distribution of ligaments’ ossification by extracranial or intracranial location and partial or complete ossification type is summarized in Tables 3, 4. A significant PTAB predominance (*P* < 0.001) was identified between the PTAB and PTSB existence, as well as a CCLB predominance between the CCLB and AICLB and between the CCLB and PICLB existence. The gender and age impact on the ECRLs and ICRLs ossification is summarized in Tables 5 and 6. ECRBs were observed in 42.3% of the male and in 41.1% of the female skulls with no gender impact (*P* = 0.899). The higher number of the ICRBs was predominantly identified in 31.1% of the male skulls (*P* = 0.048), compared to the female skulls (15.7%). Most of the ECRBs (52.8%) were identified at the age of 60 years and above, with a significant difference (*P* = 0.028), compared to the other two groups. No age impact was observed in the detection of the ICRBs (*P* = 0.713).

**Table 1:** Laterality in the presence of the sphenoid bone (SB) extracranial and intracranial ossified ligaments (ECRLs and ICRLs), according to their ossification pattern (isolated or mixed)

Sphenoid bone ligaments’ ossification	Ossification pattern (156 skulls)			
	Isolated *n* (%)	Mixed *n* (%)	Total *N* (%)	*P*-value
ECRLs		PTAL and PTSL		
PTAL	42 (26.92)	9 (5.77)	51 (32.69)	< 0.001*
PTSL	16 (10.26)		25 (16.03)	0.230

ICRLs	Isolated *n* (%)	Mixed *n* (%)				
		CCLL and PICLL	CCLL and AICLL	CCLL and AICLL and PICLL	Total *N* (%)	*P*-value
CCLL	24 (15.38)	8 (5.13)	5 (3.21)	1	38 (24.36)	< 0.001*
AICLL	Isolated *n* (%)	AICLL and CCLL	AICLL and CCLL and PICLL			
AICLL	1 (0.64)	5 (3.21)	1 (0.64)		7 (4.49)	0.125
PICLL	Isolated *n* (%)	PICLL and CCLL	PICLL and CCLL and AICLL			
PICLL	1 (0.64)	8 (5.13)	1 (0.64)		10 (6.41)	0.021*

PTAL: pterygoalar ligament; PTSL: pterygospinous ligament; CCLL: caroticoclinoid ligament; PICLL: posterior interclinoid ligament; AICLL: anterior interclinoid ligament; *n* number of skulls, *N* total number, with bold letters (*) appear the statistically significant values performed with McNemar test, (%) prevalence of appearance

**Table 2:** Laterality (unilateral or bilateral side) in the mixed ossification pattern of the sphenoid bone (SB) extracranial and intracranial ossified ligaments (ECRLs and ICRLs)

Mixed ossification pattern of ICRLs and ECRLs	Total *N*	Unilateral *n*	Bilateral *n*
	17		
Coexistence of three ICRLs			
AICLL + PICLL (same side) and CCLL + AICLL + PICLL (contralateral)	1		
Coexistence of two ligaments (ICRL and ECRL)			
CCLL + PTAL (same side)	5	3	2
PICLL + PTSL (same side)	1	1	
CCLL + PTSL (same side)	1	1	
Coexistence of three ligaments (two ICRLs and an ECRL)			
CCLL + AICLL + PTAL (same side)	2	1	1
CCLL + PICLL + PTAL (same side)	1		1
CCLL + PICLL + PTSL (same side)	1		1
Coexistence of four ligaments (two ICRLs and two ECRLs)			
CCLL + AICLL + PTAL + PTSL (same side)	1		1
CCLL + PTAL (same side) and CCLL + PTAL + AICLL (contralateral)	1		
CCLL + PTAL (same side) and CLLL + PTAL + PICLL (contralateral)	1		
CCLL + PTAL + AICLL (same side) and CCLL + AICLL + PTAL + PTSL (contralateral)	1		
CCLL (contralateral) + PTSL (same side)	1		

PTAL: pterygoalar ligament; PTSL: pterygospinous ligament; CCLL: caroticoclinoid ligament; PICLL: posterior interclinoid ligament; AICLL: anterior interclinoid ligament; *n* number of skulls; *N *total number

**Table 3:** Distribution of the sphenoid bone (SB) extracranial ligaments’ (ECRLs) ossification according to their location (unilaterally, or bilaterally, right, or left side)

ECRLs ossification	By skull (156)	By side (312)			
		Right	Left	Total *N* (%)	*P*-value
		39 (12.5)	38 (12.18)	77 (24.68)	1.000

By ossified ligament	Unilateral *n* (%)	Bilateral *n* (%)	Total *N* (%)	*P*-value	Right	Left	Total *N* (%)	*P*-value
PTAL ossification	40 (25.64)	11 (7.05)	51 (32.69)	< 0.001*	36 (11.53)	30 (9.61)	66 (21.15)	**0.511**
Partial	32 (20.51)	9 (5.77)	41 (26.28)	< 0.001*	27 (8.65)	25 (8.01)	52 (16.66)	0.864
Complete	8 (5.13)	2 (1.28)	10 (6.41)	0.039*	9 (2.88)	5 (1.6)	14 (4.48)	0.364
PTSL ossification	21 (13.46)	4 (2.56)	25 (16.03)	< 0.001*	13 (4.17)	17 (5.45)	30 (9.61)	0.664
Partial	18 (11.54)	1 (0.64)	19 (12.18)	< 0.001*	9 (2.88)	11 (3.52)	20 (6.41)	0.815
Complete	3 (1.92)	3 (1.92)	6 (3.85)	**1.000**	4 (1.28)	6 (1.92)	10 (3.21)	0.625

*n* number of skulls; *N* total number, in bold letters (*) appear the statistically significant values (after their calculation with McNemar test); PTAL: pterygoalar ligament; PTSL: pterygospinous ligament

**Table 4:** Distribution of the sphenoid bone (SB) intracranial ligaments’ (ICRLs) ossification according to their location (unilaterally, or bilaterally, right, and left-sided),

ICRLs ossification	By skull (*N* = 156)				By side (*N* = 312)			
					Right *n* (%)	Left *n* (%)	Total *N* (%)	*P*-value
					37 (11.86)	28 (8.97)	65 (20.8)	0.035*

By ossified ligament	Unilateral *n* (%)	Bilateral* n* (%)	Total *N* (%)	*P*-value	Right *n* (%)	Left *n* (%)	Total *N* (%)	*P*-value
CCLL ossification	16 (10.27)	22 (14.10)	38 (24.36)	0.418	35 (11.22)	27 (8.65)	62 (19.87)	**0.057**
Partial	10 (6.42)	17 (10.89)	27 (17.30)	0.345	26 (8.3)	19 (6.09)	45 (14.42)	0.065
Complete	6 (3.85)	5 (3.21)	11 (7.06)	0.774	9 (2.88)	8 (2.56)	17 (5.45)	1.000
AICLL ossification	5 (3.21)	2 (1.28)	7 (4.48)	0.453	7 (2.24)	3 (0.96)	10 (3.21)	0.125
Partial	1 (0.64)	1 (0.64)	2 (1.28)	1.000	2 (0.64)	2 (0.64)	4 (1.28)	1.000
Complete	4 (2.57)	1 (0.64)	5 (3.20)	0.375	5 (1.60)	1 (0.32)	6 (1.92)	0.125
PICLL ossification	5 (3.21)	5 (3.21)	10 (6.41)	1.000	9 (2.88)	7 (2.24)	16 (5.12)	0.687
Partial	3 (1.93)	5 (3.21)	8 (5.13)	0.727	7 (2.24)	6 (1.92)	13 (4.17)	1.000
Complete	2 (1.28)	0	2 (1.28)	–	2 (0.64)	1 (0.32)	3 (0.97)	1.000

*n* number of skulls, *N* total number, with bold letters (*) appear the statistically significant values (after their calculation with McNemar test); CCLL: caroticoclinoid ligament; AICLL: anterior interclinoid ligament; PICLL: posterior interclinoid ligament

**Table 5:** Gender and age impact on sphenoid bone (SB) extracranial ligaments’ (ECRLs) ossification, in total and per each ligament (PTAL—pterygoalar, and PTSL—pterygospinous)

ECRLs ossification	By gender (129 skulls)				By age (110 skulls)				
	M (78) % (*n*)	F (51) % (*n*)	Total % (*n*)	*P*-value	20–39 (33 skulls) % (*n*)	40–59 (24) % (*n*)	60 and above (53) % (*n*)	T % (*n*)	*P*-value
ECRLs in total	42.31 (33)	41.17 (21)	41.86 (54)	0.899	30.3 (10)	25 (6)	52.83 (28)	40 (44)	0.028*

By ligament	M (78) % (*n*)	F (51) % (*n*)	Total % (*n*)	*P*-value	20–39 (33) % (*n*)	40–59 (24) % (*n*)	60 and above (53) % (*n*)	Total % (*n*)	*P*-value
PTALs in total	37.18 (29)	29.41 (15)	34.11 (44)	0.363	27.27 (9)	25 (6)	39.62 (21)	32.72 (36)	0.326
PTALs unilaterally	28.21 (22)	25.49 (13)	27.13 (35)	0.735	18.18 (6)	25 (6)	32.07 (17)	26.36 (29)	0.359
PTALs bilaterally	8.97 (7)	3.92 (2)	6.97 (9)	0.271	9.09 (3)	(0)	7.54 (4)	6.36 (7)	0.338
PTSLs in total	8.97 (7)	21.57 (11)	13.95 (18)	0.044*	9.09 (3)	8.33 (2)	18.87 (10)	13.63 (15)	0.304
PTSLs unilaterally	7.69 (6)	17.65 (9)	11.63 (15)	0.085	6.06 (2)	4.16 (1)	16.98 (9)	10.9 (12)	0.140
PTSLs bilaterally	1.28 (1)	1.96 (1)	1.55 (2)	0.760	(0)	4.16 (1)	1.89 (1)	1.81 (2)	0.508

The age (in years) was expressed in three age groups, *n* number of skulls, with bold letters (*) the statistically significant value performed after the Chi-square test; M: males; F: females; T: total

**Table 6:** Gender and age impact on sphenoid bone (SB) intracranial ligaments’ (ICRLs) ossification, in total and per each ligament

ICRLs ossification	By gender (129 skulls)				By age (110 skulls)				
	M (78) *n* (%)	F (51) *n* (%)	Total *n* (%)	*P*-value	20–39 (33) % (*n*)	40–59 (24) % (*n*)	60 and above (53) % (*n*)	Total % (*n*)	*P*-value
ICRLs in total	30.76 (24)	15.68 (8)	24.80 (32)	0.048*	24.24 (8)	29.17 (7)	20.75 (11)	23.63 (26)	0.713

By ligament’s ossification	M (78) *n* (%)	F (51) *n* (%)	Total *n* (%)	*P*-value	20–39 (33) % (*n*)	40–59 (24) % (*n*)	60 and above (53) % (*n*)	Total % (*n*)	*P*-value
CCLLs in total	30.76 (24)	13.72 (7)	24.03 (31)	0.027*	21.21 (7)	29.17 (7)	20.75 (11)	22.72 (25)	0.695
CCLLs unilaterally	12.82 (10)	3.92 (2)	9.30 (12)	0.089	9.09 (3)	16.66 (4)	5.66 (3)	9.09 (10)	0.298
CCCLs bilaterally	17.94 (14)	9.80 (5)	14.73 (19)	0.202	1.21 (4)	12.5 (3)	15.09 (8)	13.63 (15)	0.911
AICLLs in total	2.56 (2)	5.88 (3)	3.87 (5)	0.340	9.09 (3)	4.16 (1)	1.88 (1)	4.54 (5)	0.295
AICLLs unilaterally	2.56 (2)	3.92 (2)	3.10 (4)	0.673	9.09 (3)	4.16 (1)	(0)	3.63 (4)	0.083
AICLLs bilaterally	(0)	1.96 (1)	0.77 (1)	0.217	(0)	(0)	1.88 (1)	0.9 (1)	0.587
PICLLs in total	3.84 (3)	3.92 (2)	3.87 (5)	0.994	6.06 (2)	4.16 (1)	3.77 (2)	4.54 (5)	0.864
PICLLs unilaterally	1.28 (1)	(0)	0.77 (1)	0.414	3.03 (1)	(0)	(0)	0.9 (1)	0.297
PICLLs bilaterally	2.56 (2)	3.92 (2)	3.10 (4)	0.673	3.03 (1)	4.16 (1)	3.77 (2)	3.63 (4)	0.978

The age (in years) was expressed in three age groups, *n* number of skulls, with bold letters (*) the statistically significant value after the performance of the Chi-square test; M: males; F: females; T: total

## Discussion

### Development of the sphenoid bone (SB) ossification centers

The SB constitutes the central axis of the skull base development. The SB center (the so-called embryologically basisphenoid and postnatally body and lesser wing) is constituted by the presphenoid and post-sphenoid, that fuse at about 8 months. The “alisphenoid” postnatally refers to the greater wing and pterygoid plate. At 8 weeks, the ossification center for the greater wing appears in cartilage, and around the 8–9 gestational week develops the pterygoid plate’s ossification, and at 9 weeks begins the ossification in the lesser sphenoidal wing. By 16 weeks, an ossification center develops in the post-sphenoid [6]. The SB major part is formed by cartilage, except for the pterygoid process that is developed by intramembranous and endochondral ossification [17]. Vinkka [57] described in rats, the formation of the posterior and inferior parts of the pterygoid process by endochondral ossification and this is the location where the PTSB and PTAB arise.

### Development of the ossified sphenoid bone (SB) ligaments: etiological factors

Ligaments are dense-fibrous structures, attached to skeletal elements and transmitting mechanical forces [4, 35]. Various ligaments in the human body are ossified to a variable degree (partially or completely) [35]. Deposits of calcium and heterotopic bone formation were also described within the dura matter folds and spanned between the PCP, dorsum sella, clivus, and petrous bone [54]. The pathogenesis of bone formation in entheses is a multifactorial process [36], including the cytokines and several systemic factors, like adipokines and gut hormones, as well as local factors, such as BMP and Wnt signaling; while angiogenesis, mechanical stress, dietary habits, metabolic abnormalities (obesity) [16], and increased age [16] may also play a role [26]. The etiology of heterotopic ligament ossification is a dynamic, and highly complex tissue repair process, that includes trauma/injury, inflammation, mesenchymal stromal cell recruitment, chondrogenic differentiation and ossification formation [63]. Ligaments’ ossification includes their fibers degenerative alterations, that are associated with a significant increase in mineral content (Ca and P) and decrease in the extracellular matrix (elastin, elastin cross-links, fibrillin, collagen, and glycoprotein) [38]*.* Other authors [40] reported that aging did not affect the ICRBs morphology, as this phenomenon is not age related and depends on the SB complex embryology. From the other part, Natsis et al. [33] reported a higher and significant prevalence of the CCLB and ICLB occurrence in older age groups, only in cases of complete ossification. Aging was more strongly correlated with the AICLL complete ossification than the CCLL. Natsis et al. [35] explained the phenomenon of enthesopathy with the chondrocytes’ occurrence around the ossified area, justifying the high incidence of osseous bridging with aging. Chewing on one side has been considered as a factor responsible for the PTSL and the PTAL ossification in between the pterygoid muscles’ fibers [9].

### Prevalence of the ossified extracranial and intracranial ligaments (ECRLs and ICRLs)

In the current study, the SB ossified ligaments were identified in 57.05%, isolated ECRBs or ICRBs in 46.15%, and combined in 10.89%. This study identified the ECRBs in a significantly higher percentage (43.3%) compared to the ICRBs (25.64%). A significantly higher incidence of unilateral than bilateral occurrence in the ECRBs’ presence was identified. The most ossified ligament in the order of decreased frequency was the extracranial PTAL (32.69%), followed by the intracranial CCLL (24.36%), the extracranial PTSL (16.03%), and the intracranial PICLL (6.41%) and AICLL (4.49%). A significant difference was identified between the PTAB and PTSB existence, as well as between CCLB and AICLB and between CCLB and PICLB, by the present study. Nikolova et al. [37] in a Bulgarian population identified the CCLB as the most common type of the sphenoid bridging (32.6%), followed by the PTSB (12.4%) and the PTAB (2.4%). Other authors investigated exclusively the ECRBs or ICRBs; thus, no further comparison can be made.

### Prevalence of the extracranial ligaments’ (ECRLs) ossification

In this study, the most ossified ECRL was the PTAL (32.7%) followed by the PTSL (16.03%). Nikolova et al. [37] found among the ECRBs, the PTSB the most occurred. Natsis et al. [34] identified a PTAB in 31.7% in a Greek population, results close to the findings of the current study (32.7%). Lower prevalence was reported in a Brazilian (2.73%) [47] and a Kenyan (8.4%) population [48]. In the current study, the PTSB presence was recorded in 16.03%, a finding similar to Goyal and Jain [21] results who identified the PTSB presence in 17.33% in an Indian population. Another study performed in a different Greek population, identified the PTSB in 38% [2]. The lowest prevalence of the PTSB existence (0.95%) was identified by Krupanidhi et al. [28] in an Indian population.

### Prevalence of the intracranial ligaments’ (ICRLs) ossification

The most ossified ICRL in the order of decreased frequency was the CCLL (24.36%), followed by the PICLL (6.41%) and the AICLL (4.49%). Natsis et al. [33], investigating the ICRLs’ ossification, in another sample of Greek skulls found the CCLL the most ossified (60.15%), followed by the AICLL (19.5%) and the PICLL (2.4%), contrariwise to this study, in which the PICLL was identified most ossified than the AICLL. Özdoğmuş et al. [40] in Turks, identified a high incidence of 45% for the CCLBs, and an incidence of 6% for the ICLBs. Keyes [27] identified a higher prevalence of the CCLBs (27.46%) compared to the ICLBs (8.68%), similar to Inoue et al. [23] who found the relative prevalence in 36% and 4%, respectively. Skandalakis et al. [52] in their meta-analysis identified the CCLBs’ pooled prevalence in 32.6%.

### Laterality on extracranial ligaments’ (ECRLs) ossification

In this study, a partial PTAB was predominantly identified unilaterally (21.79%) than bilaterally (5.77%). The complete PTAB was predominantly identified unilaterally (6.42%) than bilaterally (1.28%). Although the partial PTSB was predominantly identified unilaterally (11.54%) than bilaterally (0.64%), the complete PTSB presented no laterality (2.56% unilaterally and 1.92% bilaterally). Pekala et al. [42] found the pooled prevalence of the partial PTAB in 8.4% and of the complete in 4.4%. Rosa et al. [46], in a Brazilian population, identified the highest prevalence of a partial PTAB (49.4%) compared to the complete PTAB (12.9%). Henry et al. [22], identified the partial and the complete PTSB pooled prevalence in 11.6% and 4.4%, respectively. In this study, the PTAB had a significant unilateral predominance, like in Pekala et al. [42] meta-analysis, in which the commonest side of PTAB occurrence was the left side.

### Laterality on intracranial ligaments’ (ICRLs) ossification

In this study, the CCLB was identified in 14.10% bilaterally and in 10.27% unilaterally. Natsis et al. [33], in another Greek sample, identified the CCLB unilateral presence in a higher prevalence (31.7%) compared to the bilateral (28.45%). The majority of the published studies pointed out a higher prevalence for the CCLB unilateral ossification compared to the bilateral, except for this study and the studies of Azeredo et al. [5], Deda et al. [12], Boyan et al. [7], and Sanobar et al. [49]. In our sample, the PICLB was identified in 5% unilaterally and bilaterally, per each and the AICLB in 3.21% unilaterally and in 1.28% bilaterally. A right-sided predominance (11.85%) was identified in ICLBs existence, compared to the left-sided (8.97%) by this study.

### Gender and age impact on extracranial ligaments’ (ECRLs) ossification

In this study, ECRBs were not affected by the gender (presence in 42.3% of the male and in 41.1% of the female skulls). Contrariwise, Henry et al. [22] reported gender impact on the PTSB presence. This study also recorded a higher significant number of ECRBs (52.8%) in skulls at the age of 60 years and above, similar to Natsis et al. [34] who identified the age impact on the PTAB existence.

### Gender and age impact on intracranial ligaments’ (ICRLs) ossification

In this study, the higher number of the ICRBs was identified in males (31.1%), compared to females (15.7%), contrariwise to Gibelli et al. [20] who reported no gender difference neither for sella turcica bridging nor for the ossified CCLLs. The current study agrees with that of Gibelli et al. [20] only for the lack of the gender impact on the CCLB presence, while Natsis et al. [33] supported a gender dimorphism in the occurrence of a partial CCLB. No age impact was detected by the present study in the ossified ICRLs, while Natsis et al. [33] identified age impact only in the completely ossified CCLLs and AICLLs. Gibelli et al. [20] identified a significant correlation of the sella bridging with the age (*P* = 0.007).

### A mixed pattern of sphenoid bone (SB) ligaments’ ossification

In this study, isolated and combined ossified ligaments were identified, depending on the location of the ECRLs and ICRLs ossification. The CCLB coexisted with other ossified bars in a higher percentage (8.33%), compared to the PTAB, PTSB, and PICLB that coexisted in 5.77% and the AICLB that coexisted with other ossified bars in 3.85%. Touska et al. [55] identified the ossification of more than one ligament in 26.7% of the patients. Most of them (76.6%) had a combination of two ossified ligaments, 23.4% had a combination of more than two ligaments and 3.1% a combination of more than three ligaments. Iwanaga et al. [24] identified the coexistence of PTSB and PTAB in 10%. In this study, one skull was identified with a mixed pattern of ossification for both the AICLL and the PICLL, similar to Archana et al. [3] type II, who identified the combined ossification of the AICLL and PICLL in 5.6%. In Gibelli et al. [20] study, the two ossified variants (CCLBs and ICLBs) were often associated, as patients with no ossified sellar ligaments, usually did not present CCLBs (*P* < 0.001). Ossified variants among similar populations suggest that excluding racial/ethnic differences, other parameters, such as gender, age, geographical distribution, and genetic and molecular factors could play an important role in ligaments’ ossification [7].

### The clinical impact of the ossified bars’ existence

SB (partial or complete) ECRLS or ICRLs ossification may entrap and compress the passing neurovascular structures causing mechanical irritation, vessels’ occlusion, and obstruction of surgical pathways [37]. The osseous bars may obstruct cranial foramina (e.g., FO) and form barriers (e.g., ICLBs or CCLBs) than hinder trans-sphenoidal surgery. In cases of combination of such accessory osseous formations with atypical adjacent foramina (of a variant morphology, i.e., of an extensive size or an atypical shape or both), the skull base imaging is essential to reveal typical or variable anatomy of the area. The gold standard imaging tool remains the 3DCT scan [30, 56, 60], offering a detailed depiction, and permitting the meticulous registration of the complicated (typical and variant) anatomy of the sella, essential for the identification of the ICA pathology [14]. Moreover, the ITF tissues’ depiction by this tool, and the use of neuro-navigation helps the FO approach, with accuracy and safety [19, 56]. Liu et al. [31] confirmed better clinical outcomes, lower recurrence rates, and shorter operation time when assisting from the guidance technique during FO radiofrequency thermocoagulation. The identification of the SB partial or complete ECRLs ossification may force radical modifications in selecting optional trajectory to access safely the FO, as their topography across the FO can significantly hinder the surgical procedure [60]. Otherwise, a wrong trajectory can lead to injury of the vital anatomical structures, like the ICA, the internal jugular vein, the eustachian tube, the ocular motor nerves (III, IV, and VI), and other neurovascular structures running to the orbit [15, 44, 60].

The extracranial complete PTAB is of greater clinical importance compared to the complete PTSB [46], due to its lateral location in relation to the FO and greater thickness (4 mm according to Chouke and Hodes [10]) comparing to the PTSB which is thinner and located medially to the FO, and thus do not create a barrier for the needle to be inserted through its lumen. The PTAB may compress on the trigeminal nerve’s mandibular division [2, 53] causing chewing disorders, pain, and numbness of the buccal area and tongue and parotid gland salivatory changes [43]. The PTAB may also hinder the transoval approaches for the treatment of trigeminal neuralgia [29]. In such difficult cases, the surgeon cannot reach FO during percutaneous procedures after repeated attempts in different angles [62]. Matys et al. [32] suggested that when the anterior access of the FO was blocked by a PTAB, the FO could be alternatively approached from an inframandibular direction. Additionally, the neurovascular structures perforating FO (the mandibular and the superficial lesser petrosal nerves, the accessory meningeal artery, and an emissary vein connecting with the pterygoid venous plexus) may be injured, resulting in neuropathy and postoperative hemorrhage, in the ITF [44].

The PTSB presence is of importance during retropharyngeal and parapharyngeal surgery and in anesthetic blockade, as may act as a barrier to the needle’s passage through FO [45]. In cases of a PTAB and a PTSB coexistence, a different treatment approach is selected, using the microvascular decompression or the stereotactic radiosurgery.

The PTAB coexistence with a FO variant in shape and size, in cases of obstruction, poses a risk of inadvertent cannulation of the foramen lacerum [15]. Coincidence of a small and narrow FO, with an ossified PTAL, a FO atypical orientation and an abnormal basicranial angulation can be troubleshooting for performing FO standard canulation, or effective percutaneous rhizotomy of the trigeminal nerve [15]. In addition, the presence of a variant pterygoid process ridge may obscure the FO, rendering the FO cannulation procedure competitive [59]. Iwanaga et al. [25] pointed out the relationship in between the base of the lateral pterygoid plate posterior border with the FO and highlighted that in cases of a distance between them (distant or removed type) the failure of percutaneous procedures for treating trigeminal neuralgia is certain.

The existence of the ICLBs and CCLBs may impede surgical approaches to the paraclinoid, sellar, and parasellar regions. Their clinical impact also depends on the size, location, and type of formation (complete or incomplete). In such cases, stepwise disconnection of the bony structures of the sella is recommended to avoid destruction of the anatomical structures involved in the lesions [18, 64]*.* Thus, standard endoscopic endonasal operations perform at the sella region may require modification to minimize risk of neurosurgical complications (e.g., rupture of the paraclinoid aneurysm, ICA damage, while removal of the clinoidal meningiomas). Specifically, the CCLBs existence may cause ICA compression, tightening or stretching, resulting in an insufficient blood supply to the brain [40]. The existence of a complete CCLB may complicate the anterior clinoidectomy (necessary to expose cavernous sinus and access the ICA clinoid segment for management of aneurysms, neoplasms, and traumatic parasellar lesions) [3, 55, 65], and increase the risk of carotid laceration [23, 50], especially in cases of the ICA aneurysmal branches [39]*.* In addition, the MCP is a reliable landmark for localization of the cavernous sinus’ anteromedial roof and the transition between the ICA intracavernous and paraclinoidal segments during endoscopic endonasal approach to the sellar, parasellar, and suprasellar region [11, 18]. Divya et al. [13] pointed out the positive correlation in between the sella bridging and the existence of impacted canines and hyperdontia, thus highlighted this ossification as a diagnostic marker of the underlying dental anomalies. In complex cases of ECRLs and ICRLs ossification with an altered FO morphology and morphometry, and coexisted variants, the preoperative 3DCT scan may reveal the possible obstruction of the needle by the PTAB, as well as the asymmetry of the FO location bilaterally [15]. In addition, skull base surgeons should also consider preoperatively possible racial, gender, and age differences, especially when applying novel techniques or modifying the surgical approach in tumor resection or aneurysm repair cases in the sella [7].

### Phylogeny details on the ossified ligaments

In different species, the ossified bars present variability in their topography and morphology. Taking as example the extracranial ossification of the PTSL into a PTSB, in lemurs, it passes medial to the FO, and in pithecoids, the usually complete PTSB passes lateral to the FO. In humans and anthropoids, it is usually incomplete [51]. A wide PTSB exists in skulls of herbivores, carnivores, and mature monkeys [1], and a small one is seen in rodents. In new-world monkeys, the PTSB lacks [58] Therefore, the presence of PTSB in humans is a phylogenic remnant [1, 58]. The PTAB is normally seen in lower animals and persists in a variable percentage among different races of the human population [41].

## Study strengths and limitations

The study’s strength is the simultaneous investigation of the SB ossification extent, extracranially and intracranially, giving emphasis on each ligament, laterality, gender, and age impact. The isolated and mixed pattern of these ligaments’ ossification is also highlighted. The study’s limitations are: 1) the low number of the identified ligaments (ECRLs in 66 skulls and ICRLs in 40 skulls) that did not permit us to further investigate the gender and the age impact on each ligament’s existence, 2) the high number of broken parts in the PCPs did not permit to further correlate the ossification tendency in between the three CPs, 3) the lack of morphometric measurements, as well as the unavailability to investigate the soft tissues of the extracranial area (calculation of the laterality effect), and 4) the lack of soft tissues, thus the PTAL and PTSL variant morphology in relation to the mandibular nerve distribution could not be recorded, according to the novel classification approach of Iwanaga et al. [24].

## Conclusions

This study reveals the predominant presence of the ECRBs compared to the ICRBs. The most occurred ECRB was the PTAB and regarding the ICRB, the most occurred was the CCLB, followed by the PICLB. The ECRBs were predominantly identified unilaterally, while the ICRBs were found with a higher prevalence bilaterally, with no side predominance. ECRBs and ICRBs predominantly identified in male skulls. The ECRBs were predominantly found in skulls of 60 years and above. In most cases, isolated PTABs, PTSBs, and CCLBs were identified, contrariwise to the AICLBs and the PICLBs that occurred in combination with other ICLBs. Detailed knowledge of the typical anatomy and variant morphology of the SB extracranial and intracranial area and the ossification extent of the adjacent ligaments is essential to improve the techniques of approaches, in selection to the lesion, to avoid complications. The detailed imaging of the area may improve the accuracy and safety of manipulations (safe distance and angulation) to minimize complications.

## Data Availability

Data and material related to the report will be available with the corresponding author for further reference.
